# Fast-Acting and Receptor-Mediated Regulation of Neuronal Signaling Pathways by Copaiba Essential Oil

**DOI:** 10.3390/ijms21072259

**Published:** 2020-03-25

**Authors:** Yasuyo Urasaki, Cody Beaumont, Michelle Workman, Jeffery N. Talbot, David K. Hill, Thuc T. Le

**Affiliations:** 1 College of Pharmacy, Roseman University of Health Sciences, 10530 Discovery Drive, Las Vegas, NV 89135, USA; yurasaki@roseman.edu (Y.U.); jtalbot@roseman.edu (J.N.T.); 2 dōTERRA International, LLC, 389 South 1300 West, Pleasant Grove, UT 84062, USA; cbeaumont@doterra.com (C.B.); marndt@doterra.com (M.W.); drhill@doterra.com (D.K.H.)

**Keywords:** apoptosis, β-caryophyllene, capillary isoelectric focusing, copaiba essential oil, nanofluidic proteomics, protein post-translational modification, neuronal signaling pathways, pI3K/Akt/mTOR, JAK/STAT, MAPK

## Abstract

This study examined the biological activities of copaiba essential oil via measurement of its effects on signaling pathways in the SH-SY5Y neuronal cell line. Nanofluidic proteomic technologies were deployed to measure the phosphorylation of biomarker proteins within the signaling cascades. Interestingly, copaiba essential oil upregulated the pI3K/Akt/mTOR, MAPK, and JAK/STAT signaling pathways in neuronal cells. The effects of copaiba essential oil peaked at 30 min post-treatment, with a half-maximal effective concentration (EC_50_) of approximately 80 ng/mL. Treatment with cannabinoid receptor 2 (CB2) agonist AM1241 or the inverse agonist BML190 abrogated the regulatory effects of copaiba essential oil on the pI3K/Akt/mTOR signaling pathway. Surprisingly, copaiba essential oil also activated the apoptosis signaling pathway and reduced the viability of SH-SY5Y cells with an EC_50_ of approximately 400 ng/mL. Furthermore, β-caryophyllene, a principal constituent of copaiba essential oil, downregulated the pI3K/Akt/mTOR signaling pathway. Taken together, the findings indicated that copaiba essential oil upregulated signaling pathways associated with cell metabolism, growth, immunity, and apoptosis. The biological activities of copaiba essential oil were determined to be fast acting, CB2 mediated, and dependent on multiple chemical constituents of the oil. Nanofluidic proteomics provided a powerful means to assess the biological activities of copaiba essential oil.

## 1. Introduction

Copaiba essential oil is a concentrated liquid obtained by steam distillation of the oleoresin of the copaiba tree. Copaiba essential oil is highly versatile and broadly utilized across multiple industries, including the cosmetic, food and wellness industries [1,2]. Currently, copaiba essential oil is being used extensively for its fragrant and emollient properties in various cosmetic products. In addition, the U.S. Food and Drug Administration has approved the use of copaiba essential oil as a flavoring agent in foods and beverages [3]. Interestingly, copaiba essential oil contains complex mixtures of terpenes that exert a broad range of biological activities, including antimicrobial, antifungal, antiparasite, anti-inflammatory, and anticancer activities [2]. Notably, β-caryophyllene, a major terpene of copaiba essential oil, selectively binds to cannabinoid receptor 2 (CB2) [4]. Activation of CB2 is a therapeutic strategy for the treatment of multiple medical conditions, including pain and inflammation [5,6,7]. Recently, there has been immense interest in the therapeutic potential of copaiba essential oil and its principal constituents [8].

The use of copaiba essential oil for drug development faces numerous challenges with regard to quality control. Similar to those of many botanical extracts, the chemical profile of copaiba essential oil varies depending on the botanical species, geography, soil composition, and rainfall index [2,8]. Furthermore, copaiba oleoresin is harvested through perforation of the trunk and collection of the oleoresin that drips out. Commercially traded copaiba oleoresin is often a mixture of oleoresin from many trees and species, which complicates the botanical species identification and reduces the manufacturing precision of copaiba essential oil. A broad range of analytical methods coupled with sophisticated chemometrics have been developed to identify chemical variations in copaiba essential oil [8,9,10,11]. However, how such chemical variations affect the biological activities and the safety and efficacy profiles of copaiba essential oil remains unclear.

In this study, the biological activities of copaiba essential oil were evaluated via measurement of the effects of the essential oil on the signaling pathways in a cultured SH-SY5Y human neuroblastoma cell line. The SH-SY5Y cell line is a model system for neuronal signaling research due to its conservation of genes and pathways associated with disease pathogenesis [12]. Signaling pathways are composed of series of protein kinases that relay extracellular stimuli to trigger cellular responses. Measurement of the effects of copaiba essential oil on signaling pathways was chosen as a robust means of biological activity assessment. The effects of copaiba essential oil on SH-SY5Y cells were characterized using nanofluidic protein post-translational modification (PTM) profiling technologies, which measure changes in the PTM profiles of signaling proteins [13,14]. Briefly, total protein extracts of SH-SY5Y cells were separated by charge or size using capillary isoelectric focusing (cIEF) or Western immunoassays in matrix-filled capillaries, respectively [15]. Changes in the isoelectric points or in the binding of primary antibodies that recognize specific modification sites were used to measure protein PTMs. Previously, nanofluidic protein PTM profiling had been deployed to identify aberrant signaling pathways in diseased tissue biopsies [16,17,18,19,20,21,22] and to assess the quality of essential oils [23] and cannabidiol oils [24]. Here, nanofluidic protein PTM profiling was deployed to characterize the effects of copaiba essential oil on neuronal signaling pathways.

## 2. Results

### 2.1. Chemical Composition of Copaiba Essential Oils

**Figure 1:**
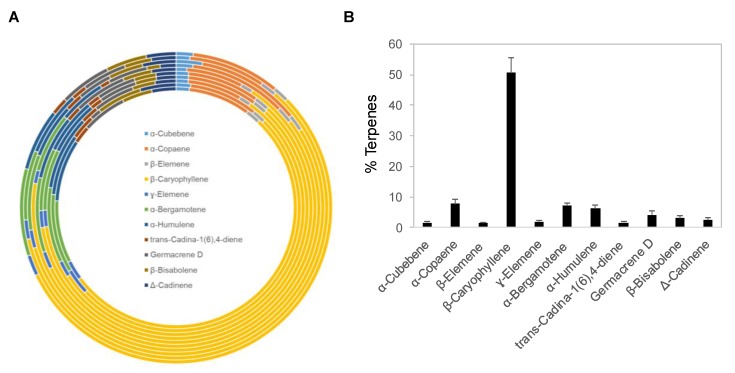
Terpene composition of copaiba essential oil. (**A**) Distribution of 11 major terpenes in 10 different batches of copaiba essential oil. Terpene composition was analyzed with gas chromatography coupled with mass spectrometry. (**B**) Percentages of 11 major terpenes in copaiba essential oil. The error bars represent the standard deviations across 10 different batches of copaiba essential oil.

Copaiba essential oil was extracted by steam distillation of oleoresins collected from *Copaifera reticulata*, *Copaifera officinalis*, *Copaifera coriacea*, and *Copaifera langsdorffii*. Ten copaiba essential oil samples with different batch numbers were analyzed with gas chromatography coupled with mass spectrometry (GC–MS) for their terpene compositions. Out of approximately 60 terpenes identified in copaiba essential oil, eleven major terpenes are summarized in Figure 1A and Table 1, including α-cubebene, α-copaene, β-elemene, β-caryophyllene, γ-elemene, α-bergamotene, α-humulene, trans-cadina-1(6),4-diene, germacrene D, β-bisabolene, and Δ-cadinene. Collectively, these eleven terpenes accounted for approximately 88% of the total terpenes in copaiba essential oil. Notably, β-caryophyllene was the dominant terpene; on average, it constituted approximately 51% of the total terpenes in copaiba essential oil (Figure 1B). Four other major terpenes, including α-copaene, α-bergamotene, α-humulene, and germacrene D, collectively accounted for approximately 25% of the total terpenes in copaiba essential oil (Table 1).

### 2.2. Time-Dependent Positive Regulation of the pI3K/Akt/mTOR Signaling Pathway

**Figure 2:**
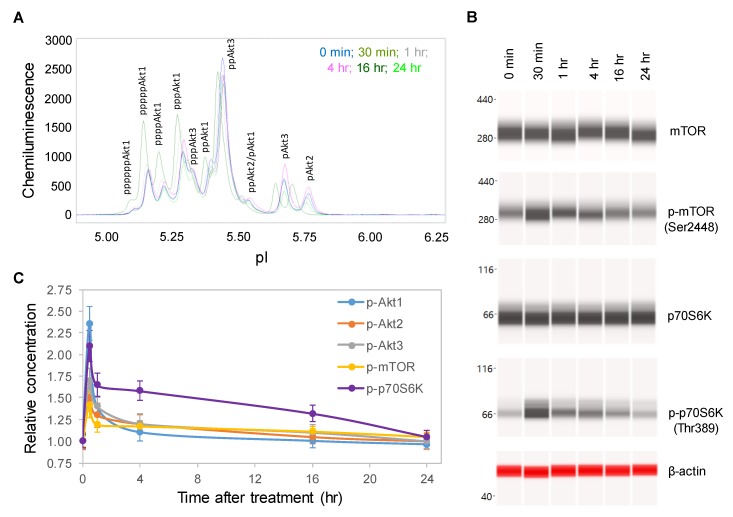
Time-dependent positive regulation of the PI3K/Akt/mTOR signaling pathway by copaiba essential oil. (**A**) Pan-Akt profiles and (**B**) expression levels of the mTOR and p70S6K phosphoisoforms in SH-SY5Y cells as functions of time after treatment. (**C**) Relative concentrations of the Akt1 (blue), Akt2 (orange), Akt3 (gray), mTOR (yellow), and p70S6K (purple) phosphoisoforms in SH-SY5Y cells as functions of time after treatment. The relative concentration describes the fold change in a protein phosphoisoform after treatment compared to the control condition. The error bars are the standard deviations of six repeated measurements per experimental condition. SH-SY5Y cells were treated with 100 ng/mL copaiba essential oil.

One random copaiba essential oil batch was selected to study its effects on the pI3K/Akt/mTOR signaling pathway, which regulates neuronal cell growth, longevity, and energy metabolism [25,26]. Following the treatment of SH-SY5Y cells with 100 ng/mL copaiba essential oil, total cell extracts were collected at specific time points and then assayed for protein PTM profiles. Specifically, the protein PTM profiles of Akt1, Akt2, Akt3, mTOR and p70S6K were measured with either cIEF or Western immunoassays. Using cIEF immunoassays, a single primary antibody against pan-Akt was sufficient to resolve the distribution of Akt1, Akt2, and Akt3 phosphoisoforms (Figure 2A and Figure S1A,B). Primary antibodies against Akt1, Akt2, or Akt3 were also used, and their phosphoisoform profiles were in agreement with those obtained with the pan-Akt primary antibody (Figure S2A–C). In addition, capillary Western immunoassays were used to measure the expression of mTOR (Ser2448) and p70S6K (Thr389) phosphoisoforms (Figure 2B). Interestingly, the levels of the phosphoisoforms of Akt1, Akt2, Akt3, mTOR, and p70S6K increased and peaked at 30 min after treatment with copaiba essential oil before steadily declining over the next 24 h (Figure 2C). Among the Akt isoforms, copaiba essential oil induced greater effects on the phosphorylation of the Akt1 isoform than on the Akt2 and Akt3 isoforms. Clearly, copaiba essential oil induced time-dependent positive regulation of the pI3K/Akt/mTOR signaling pathway in the SH-SY5Y neuronal cell line.

### 2.3. Dose-Dependent Positive Regulation of the pI3K/Akt/mTOR Signaling Pathway

**Figure 3:**
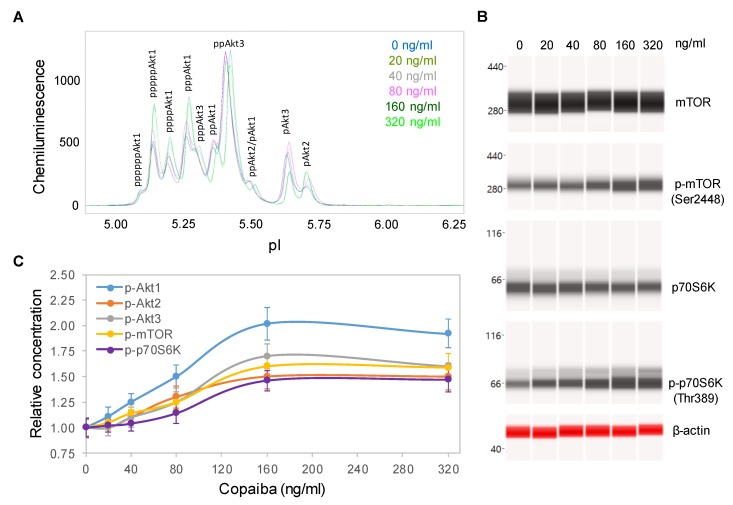
Dose-dependent positive regulation of the PI3K/Akt/mTOR signaling pathway by copaiba essential oil. (**A**) Pan-Akt profiles and (**B**) expression levels of the mTOR and p70S6K phosphoisoforms as functions of copaiba essential oil dose. (**C**) Relative concentrations of the Akt1 (blue), Akt2 (orange), Akt3 (gray), mTOR (yellow), and p70S6K (purple) phosphoisoforms as functions of copaiba essential oil dose. The relative concentration describes the fold change in a protein phosphoisoform after treatment compared to the control condition. The error bars are the standard deviations of six repeated measurements per experimental condition. SH-SY5Y cells were treated with various concentrations of copaiba essential oil for 30 min.

Dose dependency assays were performed to further examine the effects of copaiba essential oil on the pI3K/Akt/mTOR signaling pathway. SH-SY5Y cells were treated with serial dilutions of copaiba essential oil for 30 min and then assayed for the protein PTM profiles. A positive correlation between the concentration of copaiba essential oil and the phosphorylation of Akt1, mTOR, and p70S6K was observed (Figure 3A–C). The half-maximal effective concentration (EC_50_) of copaiba essential oil for activation of the pI3K/Akt/mTOR pathway was approximately 80 ng/mL. Dose dependency assays further confirmed the positive regulation of the pI3K/Akt/mTOR signaling pathway by copaiba essential oil.

### 2.4. CB2-Mediated Regulation of the pI3K/Akt/mTOR Signaling Pathway

**Figure 4:**
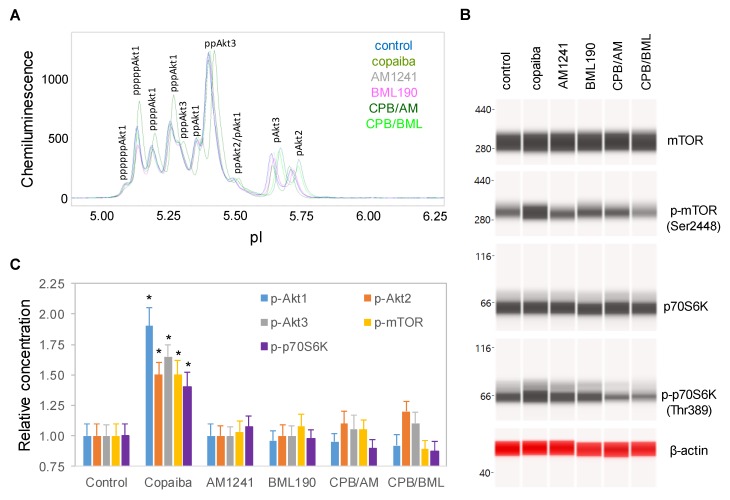
CB2 agonists abrogated the activation of the PI3K/Akt/mTOR signaling pathway by copaiba essential oil. (**A**) Pan-Akt profiles and (**B**) expression levels of the mTOR and p70S6K phosphoisoforms in SH-SY5Y cells at 30 min following treatment with copaiba essential oil (100 ng/mL), AM1241 (200 nM), BML190 (10 µM), 100 ng/mL copaiba essential oil together with 200 nM AM1241 (CPB/AM), or 100 ng/mL copaiba essential oil together with 10 µM BML190 (CPB/BML). (**C**) Relative concentrations of the Akt1 (blue), Akt2 (orange), Akt3 (gray), mTOR (yellow), and p70S6K (purple) phosphoisoforms in SH-SY5Y cells at 30 min following treatment with copaiba essential oil, AM1241, BML190, CPB/AM, or CPB/BML. The relative concentration describes the fold change in a protein phosphoisoform after treatment compared to the control condition. The error bars are the standard deviations of six repeated measurements per experimental condition. The asterisks indicate statistical significance for *p* ≤ 0.05 versus the control.

To examine the role of CB2 in the biological activities of copaiba essential oil, SH-SY5Y cells were treated with copaiba essential oil in the presence of the CB2 agonist AM1241 [27] or the CB2 inverse agonist BML190 [28]. AM1241 and BML190 served as competitors to β-caryophyllene, a major constituent of copaiba essential oil, for CB2 binding. At 30 min after treatment with copaiba essential oil, increased phosphorylation of Akt1, mTOR, and p70S6K was observed (Figure 4A–C). Treatment with AM1241 or BML190 alone had no detectable effect on the phosphorylation of Akt1, mTOR, or p70S6K. Interestingly, treatment with copaiba essential oil together with AM1241 or BML190 abrogated the positive effects of copaiba essential oil on the phosphorylation of Akt1, mTOR, and p70S6K. Our data indicated that CB2 mediated the effects of copaiba essential oil on the pI3K/Akt/mTOR signaling pathway.

### 2.5. Time-Dependent Positive Regulation of the MAPK and JAK/STAT Signaling Pathways

**Figure 5:**
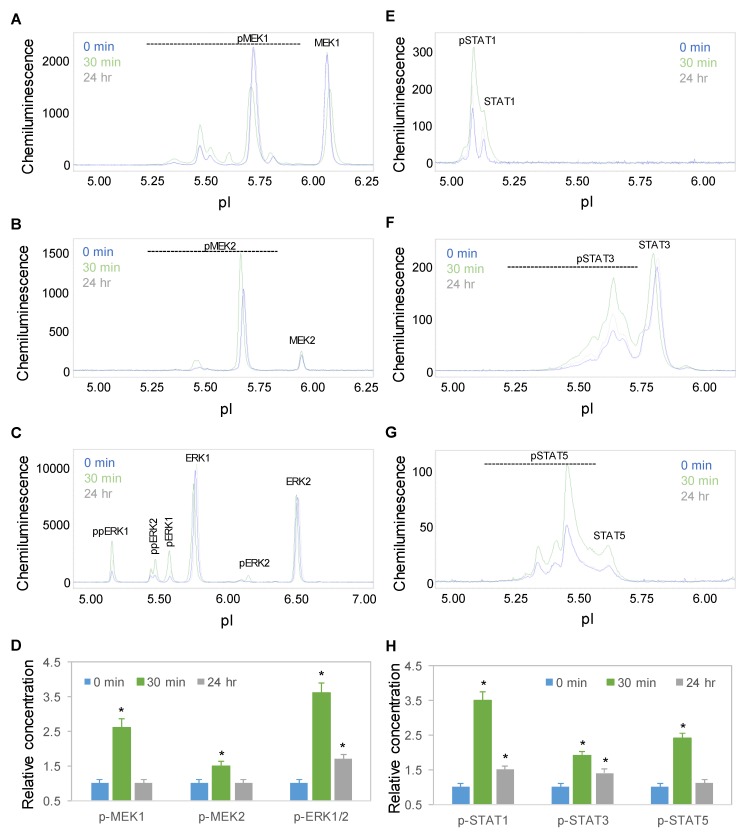
Time-dependent activation of the MAPK and JAK/STAT signaling pathways by copaiba essential oil. (**A**–**C**) MEK1 (**A**), MEK2 (**B**), and ERK1/2 (**C**) profiles as functions of time after treatment. (**D**) Relative concentrations of the MEK1, MEK, and ERK1/2 phosphoisoforms as a function of time after treatment. (**E**–**H**) STAT1 (**E**), STAT3 (**F**), and STAT5 (**G**) profiles as functions of time after treatment. (**H**) Relative concentrations of the STAT1, STAT3, and STAT5 phosphoisoforms as functions of time after treatment. The relative concentration describes the fold change in a protein phosphoisoform after treatment compared to the control condition. The error bars are the standard deviations of six repeated measurements per experimental condition. The asterisks indicate statistical significance for *p* ≤ 0.05 versus the control. SH-SY5Y cells were treated with 100 ng/mL copaiba essential oil.

Time-dependent positive regulation by copaiba essential oil was further observed for the mitogen-activated protein kinase (MAPK) and JAK/STAT signaling pathways. On the one hand, the protein PTM profiles of MEK1, MEK2, and ERK1/2 were used to measure the signaling activity of the MAPK pathway, which regulates neuronal cell proliferation and differentiation [29]. On the other hand, the protein PTM profiles of STAT1, STAT3, and STAT5 were used to measure the signaling activity of the JAK/STAT pathway, which regulates neuronal innate immunity [30]. Following treatment with 100 ng/mL copaiba essential oil, the expression levels of the MEK1, MEK2, ERK1, and ERK2 phosphoisoforms of the MAPK pathway and STAT1, STAT3, and STAT5 phosphoisoforms of the JAK/STAT pathway increased, peaked at 30 min, and then declined toward baseline levels at 24 h (Figure 5A–H). The temporal regulatory effects of copaiba essential oil on the MAPK and JAK/STAT signaling pathways mirrored those on the pI3K/Akt/mTOR signaling pathway.

### 2.6. Time-Dependent Activation of the Apoptosis Signaling Pathway

**Figure 6:**
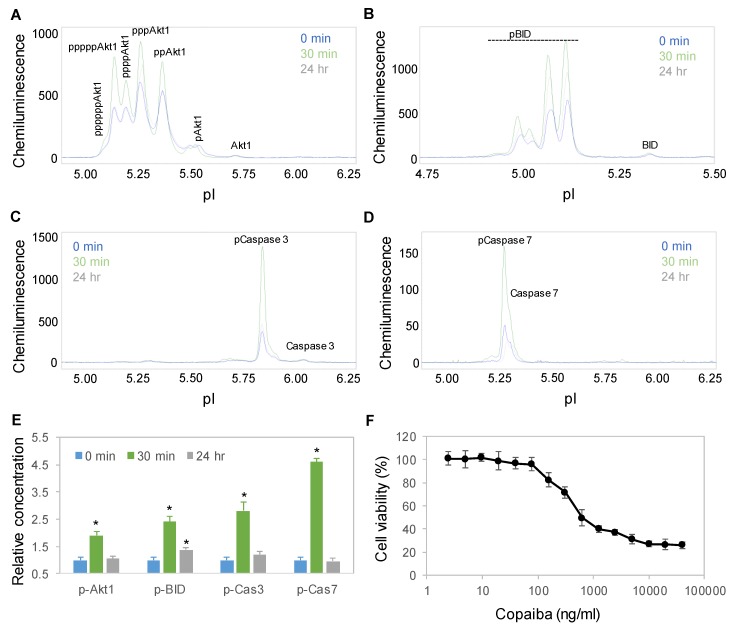
Time-dependent positive regulation of the apoptosis signaling pathway by copaiba essential oil. (**A–D**) Akt1 (**A**), BID (**B**), caspase 3 (**C**), and caspase 7 (**D**) profiles as functions of time after treatment of SH-SY5Y cells with 100 ng/mL copaiba essential oil. (**E**) Relative concentrations of p-Akt1, caspase 3, and caspase 7 as functions of time after treatment with 100 ng/mL copaiba essential oil for 0 min (blue), 30 min (orange), and 24 h (gray). (**F**) Viability percentages of SH-SY5Y cells as functions of the concentrations of copaiba essential oil. The relative concentration describes the fold change in a protein phosphoisoform after treatment compared to the control condition. The error bars are the standard deviations of six repeated measurements per experimental condition. The asterisks indicate statistical significance for *p* ≤ 0.05 versus the control.

Interestingly, treatment with copaiba essential oil also activated the apoptosis signaling pathway in SH-SY5Y cells. Apoptosis is a programmed sequence of events that lead to cell death. The PTM profiles of the Akt1, BID, caspase 3, and caspase 7 proteins were used to monitor the activity of the apoptosis signaling pathway [31]. Following treatment with 100 ng/mL copaiba essential oil, the expression levels of Akt1, BID, caspase 3 and caspase 7 phosphoisoforms increased significantly, peaked at 30 min, and then declined toward baseline levels at 24 h (Figure 6A–E). Consistently, treatment of SH-SY5Y cells with copaiba essential oil for 24 h reduced the viability of the cells with an EC_50_ of approximately 400 ng/mL (Figure 6F). The cytotoxic effects of copaiba essential oil are consistent with the reported antiproliferative activities of copaiba oleoresin and its constituents on a broad range of human cancer cell lines [2,8].

## 3. Discussion

In this study, we found that copaiba essential oil positively regulated multiple signaling pathways in neuronal cells in a time- and dose-dependent manner. The signaling pathways positively regulated by copaiba essential oil included the pI3K/Akt/mTOR, MAPK, and JAK/STAT pathways, which regulate neuronal metabolism, proliferation and immunity, respectively. The positive regulatory effects of copaiba essential oil peaked at 30 min with an EC_50_ of approximately 80 ng/mL and were mediated in part by CB2. The positive effects of copaiba essential oil on neuronal signaling pathways were consistent with its reported functions in metabolism [32], wound healing [33,34,35,36], and anti-inflammation [37,38,39,40,41]. Interestingly, copaiba essential oil also activated the apoptosis signaling pathway in a time-dependent manner and reduced the viability of neuronal cells with an EC_50_ of approximately 400 ng/mL. This observation is consistent with the reported anticancer effects of copaiba essential oil [42,43]. Future evaluation of the therapeutic potential of copaiba essential oil should take into consideration its toxicity profile.

Notably, we observed differential regulation of signaling pathways by copaiba essential oil in different cell types. Previously, we have reported that copaiba essential oil upregulates the MAPK and JAK/STAT signaling pathways in the HepG2 hepatocyte cell line [23]. The positive regulatory effects of copaiba essential oil on the MAPK and JAK/STAT signaling pathways were conserved in the SH-SY5Y neuronal cell line. In contrast, we found that copaiba essential oil had opposite regulatory effects on the pI3K/Akt/mTOR signaling pathway in the two different cell lines, causing activation in SH-SY5Y cells and suppression in HepG2 cells. The precise mechanism underlying this differential regulation in different cell types is unclear. However, we found that the composition of Akt isoforms was different between liver and brain cells. In SH-SY5Y neuronal cells, all three Akt isoforms, Akt1, Akt2, and Akt3 were present with 40%, 10%, and 50% distributions, respectively. In contrast, in HepG2 liver cells, Akt3 was completely absent, and Akt1 and Akt2 were present with 90% and 10% distributions, respectively (Figure S3). Akt isoforms have both overlapping and distinctive functional specificity and tissue distribution [44]. Akt1 regulates cell survival, growth, and proliferation; Akt2 regulates glucose metabolism; and Akt3 regulates neuronal development. In particular, Akt3 also regulates ion and cell volume homeostasis via the WNK1/SGK1 signaling pathway [45,46]. It is plausible that Akt3 is responsible for the differential regulation of the pI3K/Akt/mTOR signaling pathway in liver and brain cells, although further investigation is warranted.

Furthermore, we continued to observe differential effects on cell signaling pathways between copaiba essential oil and its principal constituent, β-caryophyllene. We have previously reported opposite regulatory effects of copaiba essential oil and β-caryophyllene on the JAK/STAT signaling pathway in HepG2 liver cells [23]. In this study, we found that treatment with isolated β-caryophyllene negatively regulated the pI3K/Akt/mTOR signaling pathway in a dose-dependent manner (Figure S4A–C). Isolated β-caryophyllene reduced the viability of SH-SY5Y cells with an EC_50_ of approximately 1.25 µg/mL, which might contribute to the observed toxicity profile of copaiba essential oil (Figure S4D). Interestingly, direct interaction with CB2 by β-caryophyllene, AM1241, or BML190 was insufficient to activate the pI3K/Akt/mTOR signaling pathway in neuronal cells (Figures S4A–C and S5A,B). The opposite effects of β-caryophyllene and copaiba essential oil on the pI3K/Akt/mTOR signaling pathway suggest a collective behavior of copaiba essential oil resulting from complex interactions of its many constituents. Indeed, evidence from the literature indicates that synergy between multiple constituents of copaiba essential oil is critical to the biological effects of the oil [47,48]. In addition to synergism, antagonism of chemical compounds also occurs in botanical extracts [49]. Future studies on individual terpenes or combinations of terpenes in copaiba essential oil could lead to in-depth understanding of their synergistic, potentiative, or antagonistic effects.

Finally, the effects of copaiba essential oil on neuronal signaling pathways described herein provide critical information for future design of therapeutic applications. First, copaiba essential oil exhibited a fast-acting mechanism, exerting maximal effects on neuronal signaling pathways within 30 min of treatment. Identification of this unique characteristic could assist in selection of dosing frequency and evaluation of the pharmacokinetics of copaiba essential oil. Second, the effects of copaiba essential oil on neuronal signaling pathways were mediated by CB2. This observation is consistent with the existing literature and supports the suitability of copaiba essential oil for pain relief and anti-inflammatory therapeutic applications [32]. Third, copaiba essential oil exerted differential effects on the pI3K/Akt/mTOR signaling pathway in different cell types. This novel observation suggests that systematic characterization of the effects of copaiba essential oil in various tissues is necessary to select the most suitable route of administration. Finally, nanofluidic protein PTM profiling permitted precise and quantitative measurement of the effects of copaiba essential oil on neuronal signaling pathways. Future integration of nanofluidic protein PTM profiling and chemical composition analysis could improve the quality control of copaiba essential oil by identifying permissible chemical variations that do not affect its biological activities. The proven capabilities of nanofluidic protein PTM profiling technology, including its ultrasensitivity, high reproducibility, high throughput and automatic operation [14,15], will be invaluable for preclinical quality testing and clinical evaluation of the pharmacodynamics of copaiba essential oil.

## 4. Materials and Methods

### 4.1. Copaiba Essential Oil

Ten copaiba essential oil batches with different batch numbers were obtained from dōTERRA International (Pleasant Grove, UT, USA). The terpene compositions of the copaiba essential oil batches are listed in Table 1. Copaiba essential oil with batch number 180437 was randomly selected to study the effects of this oil on neuronal signaling pathways.

### 4.2. GC–MS Method for Copaiba Essential Oil Analysis

The chemical composition of copaiba essential oil was analyzed using a gas chromatograph (Agilent 6890N, Santa Clara, CA, USA) coupled with a mass spectrometer (Agilent 5973 Network). The samples were loaded with an automatic injector (Agilent 7683). Sample preparation consisted of dissolving 0.02 mL of copaiba essential oil in 1.0 mL of hexane. Each sample injection was repeated three times. An Agilent DB-5MS capillary column with a length and diameter of 30 m and 0.25 mm, respectively, was used. The stationary phase film thickness was 0.25 μm. The flow rate of the carrier gas, nitrogen, was 1.2 mL/min. The temperatures of the injector, ion source, and quadrupole were 250 °C, 230 °C, and 150 °C, respectively. Samples of 3 μL were injected in split mode (40:1). The analyses were carried out in scan mode over an *m*/*z* range of 40–500. The time of analyte allocation was 52 min. The column temperature program was 40 °C for 3 min followed by 80 °C for 2 min, 120 °C for 5 min, 200 °C for 2 min, and 250 °C for another 2 min. ChemStation software was used to collect and process the data. The compounds in the samples were identified by comparing the mass spectra with standard spectra from the NIST 2014 library. Retention indices were determined. Compounds with mass spectra showing more than 95% conformity with the standard library spectra were considered. To confirm the identification, the retention indices of the analyzed compounds were compared with the literature. The relative content percentages of the analyzed compounds were based on the peak area of the total ionic current of all compounds present in each sample.

### 4.3. Cell Line and Culture Conditions

SH-SY5Y cells (cat. no. CRL2266, American Type Culture Collection, Manassas, VA, USA) were cultured in a 1:1 mixture of Eagle’s minimum essential medium (cat. no. 302003, ATCC) and F12 medium (cat. no. 302004, ATCC) supplemented with 10% fetal bovine serum (cat. no. SH30088.03, GE Healthcare Life Sciences, Pittsburgh, PA, USA).

### 4.4. Treatment Conditions

SH-SY5Y cells were grown in culture medium to approximately 70% confluence. The culture medium was replaced with culture media premixed with copaiba essential oil (100 ng/mL) or serial dilutions of copaiba essential oil, the CB2 agonist AM1241 (200 nM, Selleckchem, Houston, TX, USA), the CB2 inverse agonist BML190 (10 µM, Selleckchem), copaiba essential oil (100 ng/mL) and AM1241 (200 nM), or copaiba essential oil (100 ng/mL) and BML190 (10 µM). The incubation time in the new culture medium was used as the time after treatment. AM1241 (Ki = 3.4 nM) and BML190 (Ki = 435 nM) were used as competitors to β-caryophyllene (Ki = 155 nM) for CB2 binding [4,28,50,51]. The concentrations of AM1241 at 200 nM and BML190 at 10 µM were chosen to match or exceed that of β-caryophyllene at approximately 250 nM in 100 ng/mL copaiba essential oil. Treatment of SH-SY5Y cells with broad ranges of concentrations of AM1241 (0.1 nM – 10 µM) and BML190 (10 nM – 100 µM) were also evaluated [28,50,51]. Data on selected doses of AM1241 and BML190 are presented in the Figure S5.

### 4.5. Preparation of Cell Lysates

Approximately one million SH-SY5Y cells were incubated on ice for 10 min with 60 µL of lysis buffer (cat. no. 040-764, ProteinSimple, Santa Clara, CA, USA), sonicated for 5 s 4 times, mixed by rotation for 2 h at 4 °C, and centrifuged at 12,000 rpm in an Eppendorf 5430R microfuge for 20 min at 4 °C. The supernatant was collected as the cell lysate. The total protein concentration in the cell lysate was determined with a Bradford protein assay and adjusted to a final concentration of 0.3 µg/µL with the separation gradients (cat. no. Premix G2, pH 5–8 or pH 3–10, ProteinSimple) for charge-based cIEF immunoassays or to 0.4 µg/µL with denaturing buffers (cat. no. PS-ST01EZ or PS-ST03EZ, ProteinSimple) for size-based Western immunoassays.

### 4.6. Antibodies and Biomarker Proteins

The primary and secondary antibodies and biomarker proteins and their functions are listed in the Tables S1 and S2.

### 4.7. cIEF Immunoassays

Approximately 400 nanoliters of SH-SY5Y cell lysates in separation gradients were loaded into individual capillaries of the 96-capillary NanoPro 1000 system (ProteinSimple). Isoelectric focusing was performed at 15 mW for 50 min. The proteins were cross-linked to the capillary walls using ultraviolet irradiation for 80 s. Primary and secondary antibodies were sequentially introduced with incubation times of 120 and 60 min, respectively. Following incubation with chemiluminescence detection agents, the proteins were detected with an average exposure time of 240 s. Hsp70 was used as a loading control. All cIEF immunoassays were performed in triplicate for each protein, and duplicate experiments were performed per treatment condition, producing six repeated measurements per protein.

### 4.8. Capillary Western Immunoassays

Cell lysates in denaturing buffers were denatured at 95 °C for 5 min, and then transferred to assay plates (cat. no. SM-W004 or SM-W008, ProteinSimple) preloaded with blocking reagents, wash buffer, primary and secondary antibodies, and chemiluminescent substrates. Sized-based protein separation and detection in capillaries were performed using the default protocols of the Jess system (ProteinSimple). β-Actin was used as a loading control. All capillary Western immunoassays were performed in triplicate for each protein, and duplicate experiments were performed per treatment condition, producing six repeated measurements per protein.

### 4.9. Data Analysis

Assignment of pI values to protein phosphoisoforms was based on the literature and our own published data [18,23,24,52,53,54,55,56]. Protein expression levels were quantified using the Compass software from ProteinSimple. The expression levels of protein phosphoisoforms were adjusted with loading controls for both charge-based and size-based immunoassays, and further adjusted with the expression levels of total proteins for size-based immunoassays.

### 4.10. Cell Proliferation Assays

MTS assays (cat. no. G3581, Promega, Madison, WI, USA) were performed according to manufacturer’s protocols. MTS signals were detected with a multimode microplate reader (Synergy 2 BioTek, Winooki, VT, USA). All MTS assays were performed in triplicate with duplicate experiments per treatment condition, producing six repeated measurements per treatment condition.

### 4.11. Statistical Analysis

The data are presented as the mean values ± standard deviations across six repeated measurements. Statistical significance was calculated with Student’s t-test and thresholded at *p* ≤ 0.05 versus the control.

## Figures and Tables

**Table 1:** Terpene composition of copaiba essential oil.

	% Terpenes in Ten Different Batches of Copaiba Essential Oil									
Terpenes	%, 180437	%, 1722278	%, 182066A	%, 51911	%, 51912	%, 60326	%, 63800	%, 55172	%, 1924111	%, 73599
α-Cubebene	1.51	1.56	1.33	1.35	1.33	1.09	1.73	2.47	1.31	1.57
α-Copaene	7.19	7.43	5.97	7.73	7.74	5.83	10.09	9.84	7.36	8.24
β-Elemene	1.67	1.44	1.28	1.59	1.59	1.23	1.7	1.56	1.56	1.45
β-Caryophyllene	45.24	41.89	49.1	54.42	55	52.16	58.18	47.09	54.42	49.07
γ-Elemene	2.26	2.82	1.76	1.8	1.81	1.76	1.36	1.7	1.83	1.9
α-Bergamotene	7.19	8.51	8.3	6.66	6.72	7.68	6.17	7.12	6.67	8.07
α-Humulene	7.18	6.67	8.25	6.01	6.1	6.64	4.88	5.18	6.27	5.75
trans-Cadina-1(6),4-Diene	2.03	2.67	1.98	1.36	1.36	1.56	1.01	1.67	1.51	1.59
Germacrene D	5.16	1.2	4.57	4.15	4.23	5.45	4.12	3.28	4.29	4.76
β-Bisabolene	3.47	4.89	2.52	2.32	2.35	2.98	1.36	3.26	2.61	3.77
Δ-Cadinene	2.78	3.88	2.3	2.14	2.14	2.5	1.64	3.17	2.36	2.75

## Supplementary Materials

Supplementary materials can be found at https://www.mdpi.com/1422-0067/21/7/2259/s1. The following are available online: Supplemental Figure S1, Akt isoform identification in SH-SY5Y cell lysates using capillary isoelectric focusing (cIEF) immunoassays; Supplemental Figure S2, Short-term positive regulation of Akt isoform phosphorylation by copaiba essential oil; Supplemental Figure S3, Akt isoform identification in HepG2 cell lysates using capillary isoelectric focusing (cIEF) immunoassays; Supplemental Figure S4, Dose-dependent negative regulation of the PI3K/Akt/mTOR signaling pathway by β-caryophyllene; Supplemental Figure S5, Pan-Akt profiles of SH-SY5Y cells as functions of treatment with CB2 agonists; Supplemental Table S1, List of primary and secondary antibodies; Supplemental Table S2, List of biomarker proteins and their functions.
