# Supplementary material for: Fast-Acting and Receptor-Mediated Regulation of Neuronal Signaling Pathways by Copaiba Essential Oil
Source: Int J Mol Sci. 2020 Mar 25;21(7):2259. doi: 10.3390/ijms21072259 (PMC7177672; doi:10.3390/ijms21072259)
Supplement: Supplementary file 1 [file ijms-21-02259-s001.pdf]

Supplementary Materials

Fast-Acting and Receptor-Mediated Regulation of Neuronal Signaling Pathways by Copaiba Essential Oil

Yasuyo Urasaki[1], Cody Beaumont[2], Michelle Workman[2], Jeffery N. Talbot[1], David K. Hill[2], Thuc T. Le[1,*]

[1]College of Pharmacy, Roseman University of Health Sciences, 10530 Discovery Drive, Las Vegas, NV 89135, USA.

[2]dōTERRA International, LLC, 389 South 1300 West, Pleasant Grove, Utah 84062, USA.

*To whom correspondence should be addressed: Email: tle5@roseman.edu; Tel: 1-702-802-2820

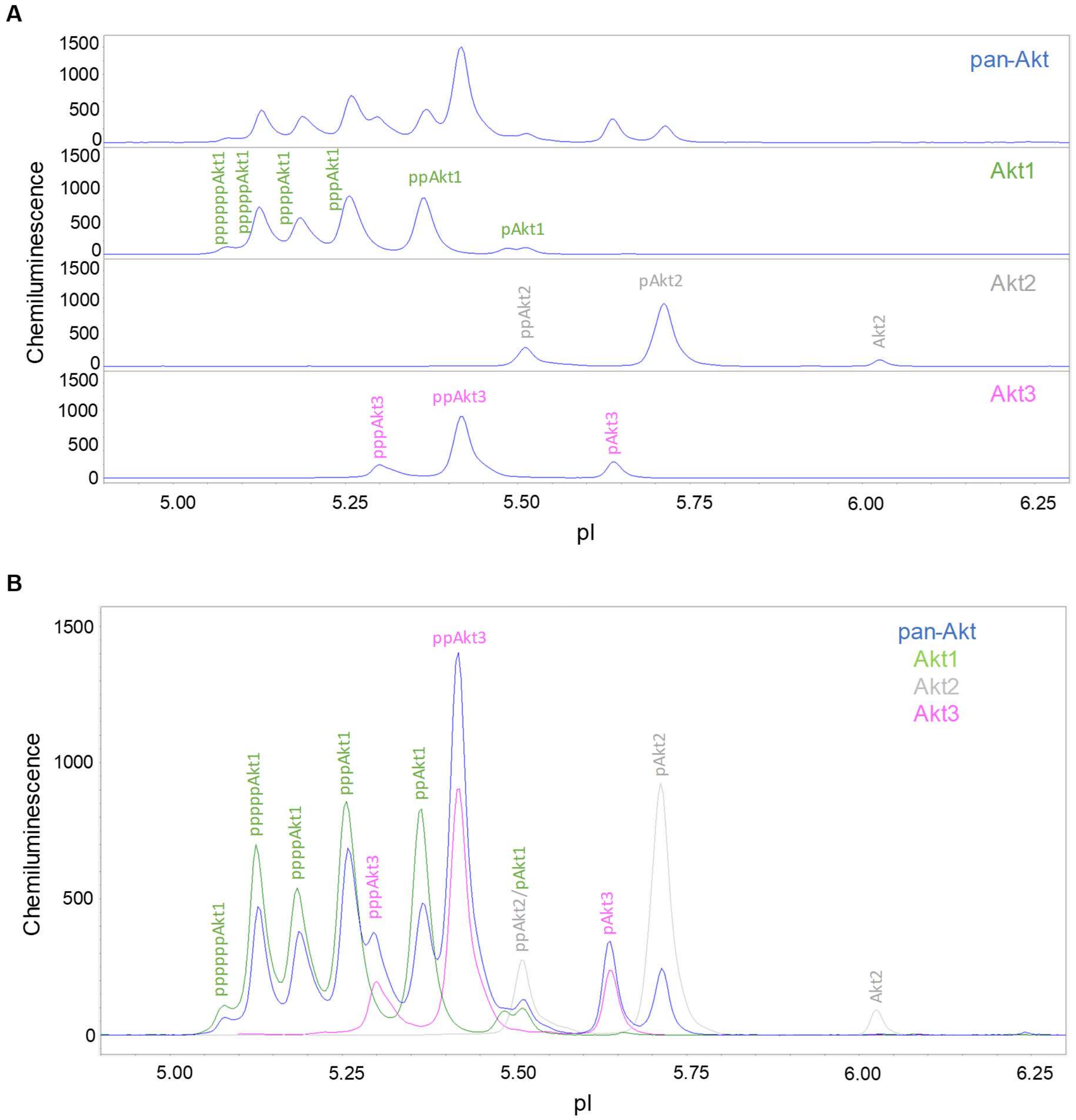

Supplemental Figure S1. Akt isoform identification in SH-SY5Y cell lysates using capillary isoelectric focusing (cIEF) immunoassays. (**A**) cIEF electropherograms of Akt profiles evaluated with pan-Akt (top panel), Akt1 (second panel), Akt2 (third panel), or Akt3 (fourth panel) primary antibodies. (**B**) Overlaid of cIEF electropherograms of Akt profiles evaluated with pan-Akt (blue), Akt1 (green), Akt2 (gray), or Akt3 (pink).

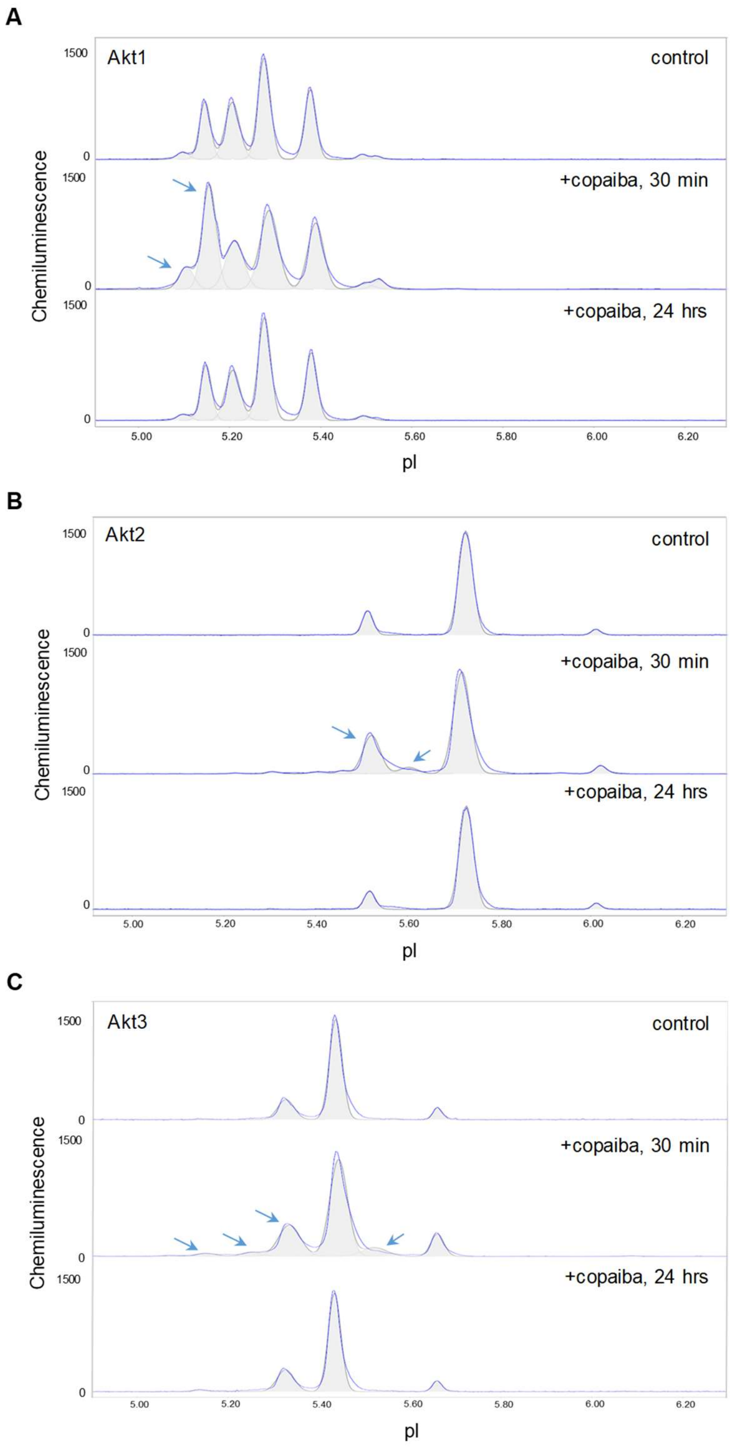

Supplemental Figure S2. Short-term positive regulation of Akt isoform phosphorylation by copaiba essential oil. Capillary isoelectric focusing electropherograms of (**A**) Akt1, (**B**) Akt2, and (**C**) Akt3 in control SH-SY5Y cells (top panel) and in SH-SY5Y cells treated with copaiba essential oil (100 ng/ml) for 30 minutes (middle panel) or 24 hours (bottom panel). Blue arrows point to changes to Akt phosphoisoforms at 30 minutes of treatment as compared with control or 24 hours of treatment.

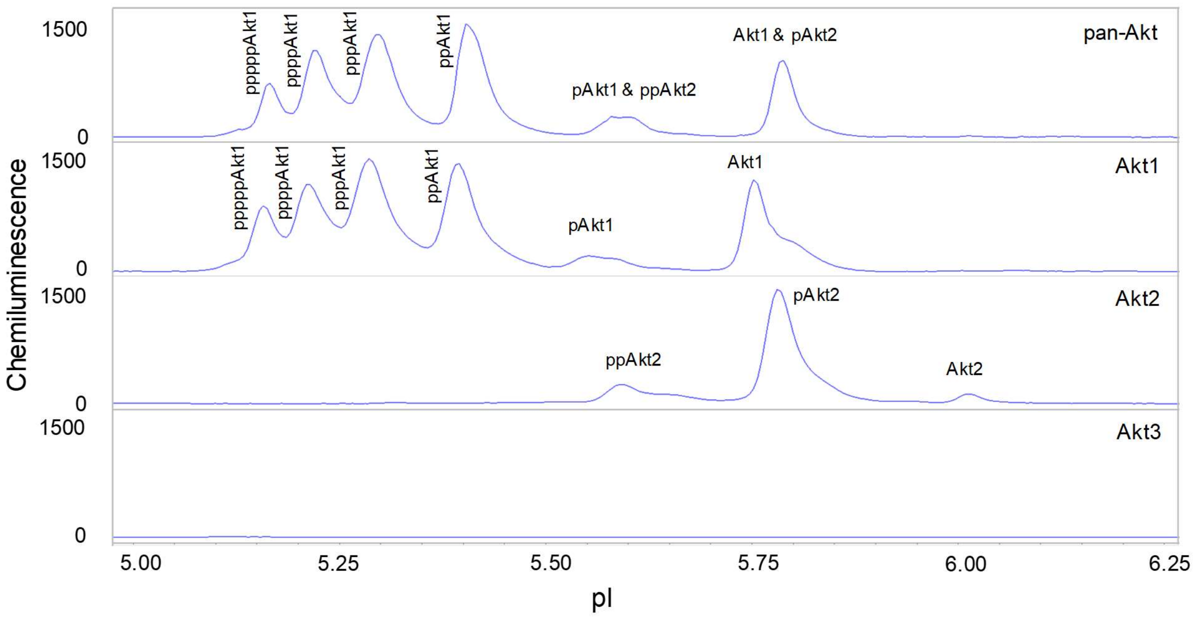

Supplemental Figure S3. Akt isoform identification in HepG2 cell lysates using capillary isoelectric focusing (cIEF) immunoassays. cIEF electropherograms of Akt profiles evaluated with pan-Akt (top panel), Akt1 (second panel), Akt2 (third panel), or Akt3 (fourth panel) primary antibodies. Note the complete absence of Akt3 in HepG2 cell lysate.

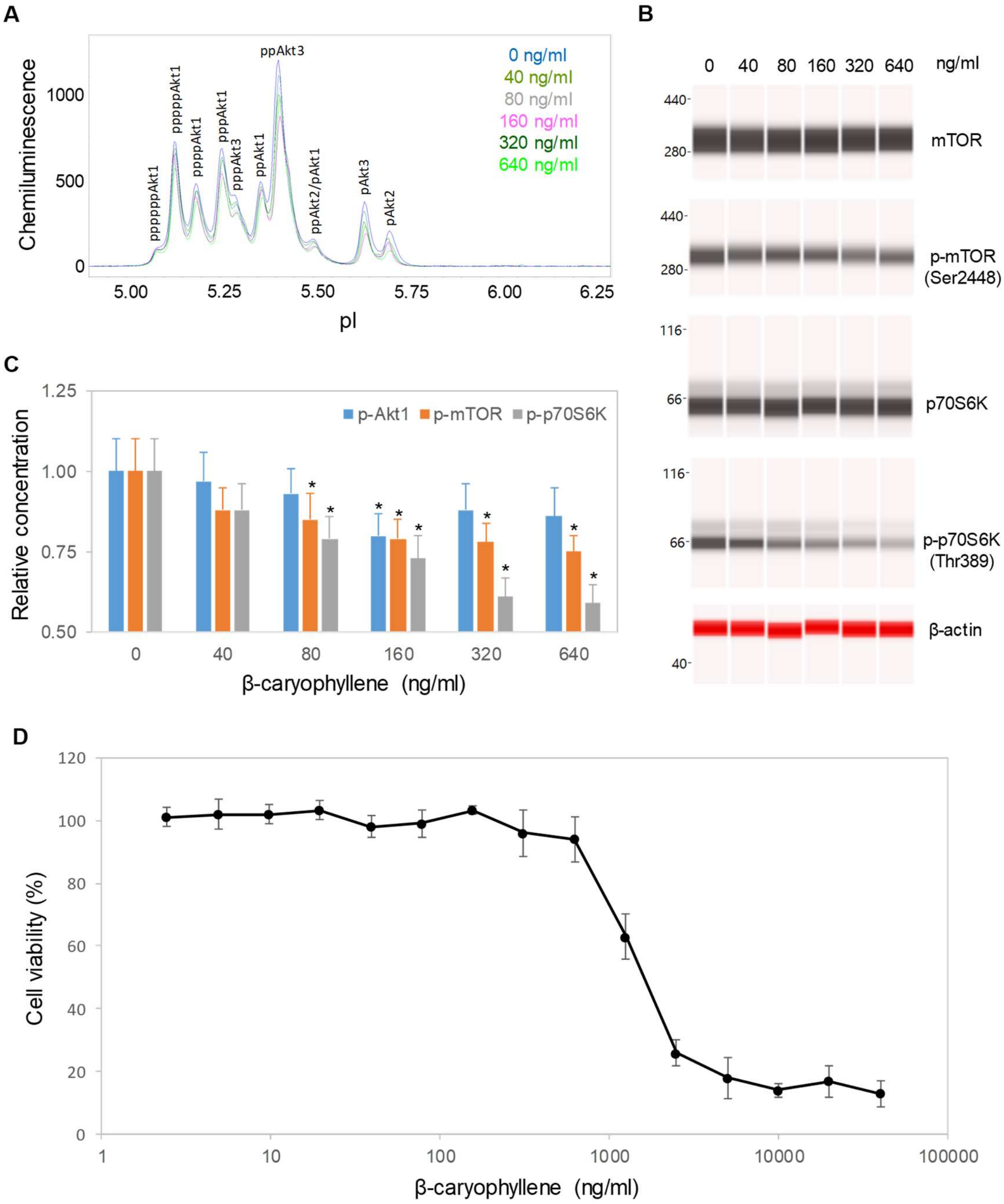

Supplemental Figure S4. Dose-dependent negative regulation of the PI3K/Akt/mTOR signaling pathway by β-caryophyllene. (**A**) Pan-Akt profiles of SH-SY5Y cells as functions of β-caryophyllene dose at 30 minutes after treatment. (**B**) Expression levels of mTOR and p70S6K phosphoisoforms as functions of β-caryophyllene dose at 30 minutes after treatment. (**C**) Relative concentrations of the Akt1 (blue), mTOR (orange), and p70S6K (gray) phosphoisoforms as functions of β-caryophyllene dose at 30 minutes after treatment. The relative concentration describes the fold change in a protein phosphoisoform after treatment compared to control condition. (**D**) The percentage viability of SH-SY5Y cells as a function of treatment with various concentrations of β-caryophyllene. The error bars are the standard deviations of six repeated measurements per experimental condition. The asterisks indicate statistical significance for $p \leq 0.05$ versus the control. β-caryophyllene was purchased from Sigma-Aldrich (cat. no. W225207, St. Louis, MO, USA).

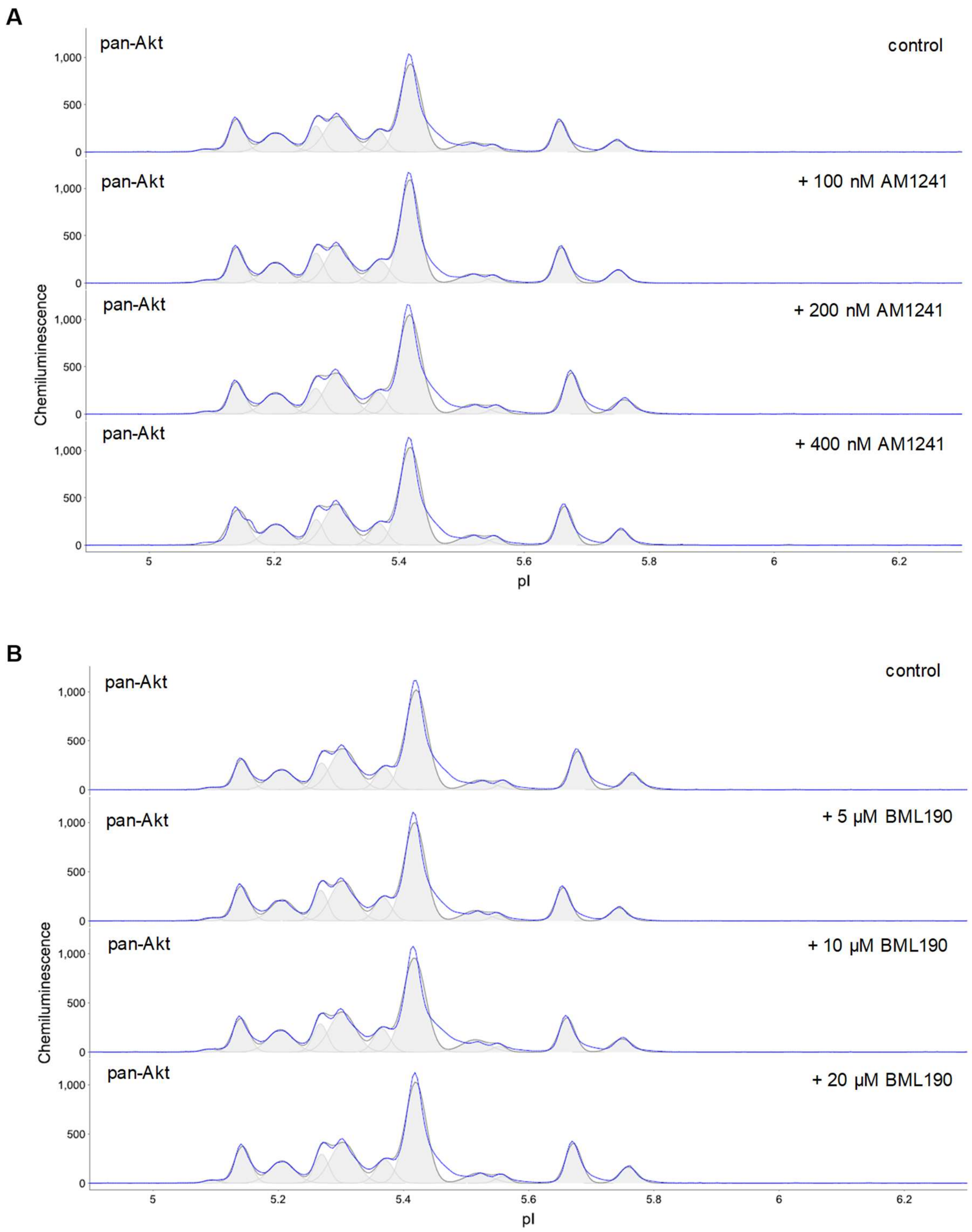

Supplemental Figure S5. Pan-Akt profiles of SH-SY5Y cells as functions of treatment with CB2 agonists. SH-SY5Y cells were treated for 30 minutes with various concentrations of (**A**) AM1241, a CB2 agonist, and (**B**) BML190, a CB2 inverse agonist. No change to the pan-Akt profile was observed following the treatment of SH-SY5Y cells with either AM1241 or BML190.

Supplemental Table S1. List of primary and secondary antibodies

№	Antibody	Cat. No.	Vendor
1	pan-Akt	8312	Santa Cruz Biotech (Dallas, TX)
2	Akt1	2938	Cell Signaling (Danvers, MA)
3	Akt2	3063	Cell Signaling
4	Akt3	8018	Cell Signaling
5	mTOR	2983	Cell Signaling
6	pmTOR (Ser2448)	5536	Cell Signaling
7	p70S6K	9202	Cell Signaling
8	pp70S6K (Thr389)	9234	Cell Signaling
9	MEK1	07-641	Millipore (Billerica, MA)
10	MEK2	9125	Cell Signaling
11	ERK1/2	040-474	Protein Simple (Santa Clara, CA)
12	STAT1	14994	Cell Signaling
13	STAT3	4904	Cell Signaling
14	STAT5	94205	Cell Signaling
15	BID	2002	Cell Signaling
16	Caspase 3	9665	Cell Signaling
17	Caspase 7	12827	Cell Signaling
18	β-actin	MAB8929	R&D Systems (Minneapolis, MN)
19	HSP60	F1800	R&D Systems
20	HSP70	4872	Cell Signaling
21	Secondary antibody (anti-rabbit HRP)	040-656	Protein Simple
22	Secondary antibody (anti-rabbit HRP)	042-206	Protein Simple
23	Secondary antibody (anti-mouse HRP)	042-205	Protein Simple
24	Secondary antibody (anti-rabbit NIR)	043-819	Protein Simple
25	Secondary antibody (anti-mouse NIR)	043-821	Protein Simple

Supplemental Table S2. List of biomarker proteins and their functions

№	Protein	Name	Function
1	Akt1	Protein kinase B, isoform 1	Apoptosis, proliferation, & cell migration
2	Akt2	Protein kinase B, isoform 2	Glucose metabolism
3	Akt3	Protein kinase B, isoform 3	Neuronal development
4	mTOR	Mechanistic target of rapamycin	Proliferation, motility, survival, autophagy,
5	p70S6K	Ribosomal S6 kinase β-1	Protein synthesis
6	MEK1	Mitogen-activated protein kinase kinase 1	Proliferation, differentiation, development
7	MEK2	Mitogen-activated protein kinase kinase 2	Proliferation, differentiation, development
8	ERK1/2	Mitogen-activated protein kinase 1/2	Proliferation, differentiation, development
9	STAT1	Signal transducer and activator of transcription 1	Immunity, proliferation, differentiation
10	STAT3	Signal transducer and activator of transcription 3	Immunity, proliferation, differentiation
11	STAT5	Signal transducer and activator of transcription 5	Immunity, proliferation, differentiation
12	BID	BH3 interacting-domain death	Pro-apoptotic member of Bcl-2 family
13	Caspase 3	Cysteine-aspartic acid protease 3	Apoptosis
14	Caspase 7	Cysteine-aspartic acid protease 3	Apoptosis
